# In vivo intervertebral disc deformation: intratissue strain patterns within adjacent discs during flexion–extension

**DOI:** 10.1038/s41598-020-77577-y

**Published:** 2021-01-12

**Authors:** Robert L. Wilson, Leah Bowen, Woong Kim, Luyao Cai, Stephanie Ellyse Schneider, Eric A. Nauman, Corey P. Neu

**Affiliations:** 1 Department of Mechanical Engineering, University of Colorado Boulder, 1111 Engineering Drive, 427 UCB, Boulder, CO 80309-0427 USA; 2 Medical Scientist Training Program, University of Colorado Anschutz, 13001 East 17th Place, Aurora, CO 80045 USA; 3 Weldon School of Biomedical Engineering, Purdue University, 206 S Martin Jischke Drive, West Lafayette, IN 47907 USA; 4 School of Mechanical Engineering, Purdue University, 585 Purdue Mall, West Lafayette, IN 47907 USA

**Keywords:** Biophysics, Biomarkers, Medical research, Biomedical engineering, Musculoskeletal system

## Abstract

The biomechanical function of the intervertebral disc (IVD) is a critical indicator of tissue health and pathology. The mechanical responses (displacements, strain) of the IVD to physiologic movement can be spatially complex and depend on tissue architecture, consisting of distinct compositional regions and integrity; however, IVD biomechanics are predominately uncharacterized in vivo. Here, we measured voxel-level displacement and strain patterns in adjacent IVDs in vivo by coupling magnetic resonance imaging (MRI) with cyclic motion of the cervical spine. Across adjacent disc segments, cervical flexion–extension of 10° resulted in first principal and maximum shear strains approaching 10%. Intratissue spatial analysis of the cervical IVDs, not possible with conventional techniques, revealed elevated maximum shear strains located in the posterior disc (nucleus pulposus) regions. IVD structure, based on relaxometric patterns of T_2_ and T_1*ρ*_ images, did not correlate spatially with functional metrics of strain. Our approach enables a comprehensive IVD biomechanical analysis of voxel-level, intratissue strain patterns in adjacent discs in vivo, which are largely independent of MRI relaxometry. The spatial mapping of IVD biomechanics in vivo provides a functional assessment of adjacent IVDs in subjects, and provides foundational biomarkers for elastography, differentiation of disease state, and evaluation of treatment efficacy.

## Introduction

The in vivo biomechanical response of biological tissues to mechanical stimuli are often indicative of health and normative function^1–3^, and yet remain largely undocumented in stiff tissues of the musculoskeletal system. Tissue stiffness is contingent on the physical and chemical composition of the tissue. The abnormal expression of these factors can alter cellular phenotype^4^, gene expression^5^, and osmotic pressure^6^ modifying tissue biomechanical properties. Quantification of biomechanical responses in stiff tissues in vivo would help inform model-based approaches as well as assist in evaluation of disease state. In vitro and ex vivo experiments are not adequate surrogates for in vivo studies as they cannot capture the complexity of tissue biomechanics in their native environments. Noninvasive imaging of soft tissue allows for the assessment and monitoring of load bearing tissues in vivo. Development of noninvasive biomechanical assessment techniques can yield greater insight into musculoskeletal tissue function both in healthy and diseased states.

The intervertebral disc (IVD) is a load bearing tissue of the musculoskeletal system that connects adjacent vertebrae in the spinal column. The IVD consists of two primary structural compartments: a compliant nucleus pulposus (NP) containing hydrophilic glycosaminoglycans (GAGs), and a stiff annulus fibrosus (AF) comprised largely of type I collagen. The IVD enables the flexibility of the spinal column within a safe range of motion while transmitting load and reducing stress from body weight and natural muscle activity within the body. Stress applied to a healthy IVD is alleviated by the ability of GAGs to retain water, increasing hydrostatic pressure and minimizing strain. In a time-dependent response, water diffuses through the concentric collagen rings of the AF when under stress^7^.

Upon trauma or overuse, the tissue can degrade and lead to intervertebral disc degeneration (IVDD), affecting approximately 25% of the global adult population^8^. IVDD can result in instability and in extreme cases severe chronic pain^9–11^. The two primary phenotypes for spinal pain, endplate-driven and AF-driven degeneration, can be distinguished by their physical origin as well as pain association with both eventuating into complete disc failure^12^. On a tissue level, later stage endplate-driven degeneration can be detected via modic changes^13^. Annulus driven degradation can be identified through tissue fissures^14^.

While the exact etiology of IVDD remains undetermined, the early stages of IVDD are characterized by a cascade of degenerative processes which occur in the extracellular matrix (ECM) and are initiated by mechanical stress confounded by genetic predisposition and lifestyle conditions. In the ECM, nucleus pulposus cell overexpression of proinflammatory cytokines and enzymes results in GAG depletion, a corresponding reduction in fixed charge density, and ultimately the loss of interstitial fluid and hydrostatic pressure in the NP which diminishes the load bearing capacity of the tissue^6^. The weakened capacity of IVD to regulate mechanical stress will result in an abnormal stress buildup leading to disc tear, bulging, or herniation^15^, the last of which can lead to global functional and local biological alterations^16^. Advanced IVD degeneration can lead to compression of spinal nerves and subsequent discogenic and radiculopathies^17^. Prior to radiographic evidence or related symptoms, the cascade of early degenerative processes at the ECM level, offer a unique window of opportunity to detect the current integrity of an IVD by characterizing the mechanical functionality of the tissue as a mechano-biomarker for diagnosis of early IVD degeneration.

Magnetic resonance imaging (MRI) is a promising modality for noninvasive soft tissue assessment and early detection of IVDD due to its superb soft tissue contrast and micron-level resolution. However, the limited sensitivity of conventional relaxometry methods (monoexponential T_2_ and T_1*ρ*_ decays) have been unable to detect the subtle macromolecular changes of early soft tissue degenerations^18,19^. Somewhat improved functional sensitivity has been achieved through endplate monitoring via a combination injection-MRI technique^20^. In contrast, elastography techniques, which noninvasively provide mechanical behaviors or parameters of materials including strain or moduli, may be superior for probing load bearing soft tissues such as the IVD^21^. Magnetic resonance elastography (MRE) has been performed on IVDs in vivo^22,23^, but predominately relies on shear wave propagation which suffer high attenuation in more rigid heterogeneous tissues (e.g. load-bearing tissues vs. heart or liver) resulting in artifacts that are difficult to minimize.

Displacements under applied loading by MRI (dualMRI), a mechano-MR imaging technique, may be an ideal technique for IVD evaluation in vivo. dualMRI provides a reasonable field of view (FOV) at micron-level resolution during application of exogenous loading to the tissue. Recently, dualMRI has been utilized to map high-resolution displacements and strain in articular cartilage under physiological loading and/or movements in vivo^24^. In vitro, dualMRI has been successfully used to characterize strain behavior in articular cartilage^25–30^ and IVD^31–33^ with high spatial resolution (100 × 100 µm^2^)^27^ and precision (11 μm displacement/0.001 strain)^28^. However, to the best of our knowledge, in vivo intradiscal strain patterns within the IVD in vivo have not been documented by directly measuring and utilizing intradisc displacements acquired via noninvasive imaging.

We document 2D voxel-level intratissue strain in vivo for multiple adjacent cervical IVDs under physiological flexion–extension using dualMRI, laying the groundwork for future studies of strain analysis in populations with severe or emerging (e.g. IVDD) pathology. In this study, we hypothesized that spatial heterogeneity in IVD strains would not relate to conventional relaxometry metrics, potentially demonstrating the need for both measures to produce greater insight into IVD integrity. To assess this hypothesis, voxel-level displacement and strain fields were calculated from dualMRI phase data acquired during cyclic flexion–extension of the cervical spine. We envision that this technique will improve IVD mathematical models and eventuate as a clinical diagnostic tool to assess IVD integrity in healthy and diseased populations, as well as provide a means to quantitatively monitor IVD treatment efficacy.

## Results

**Figure 1:**
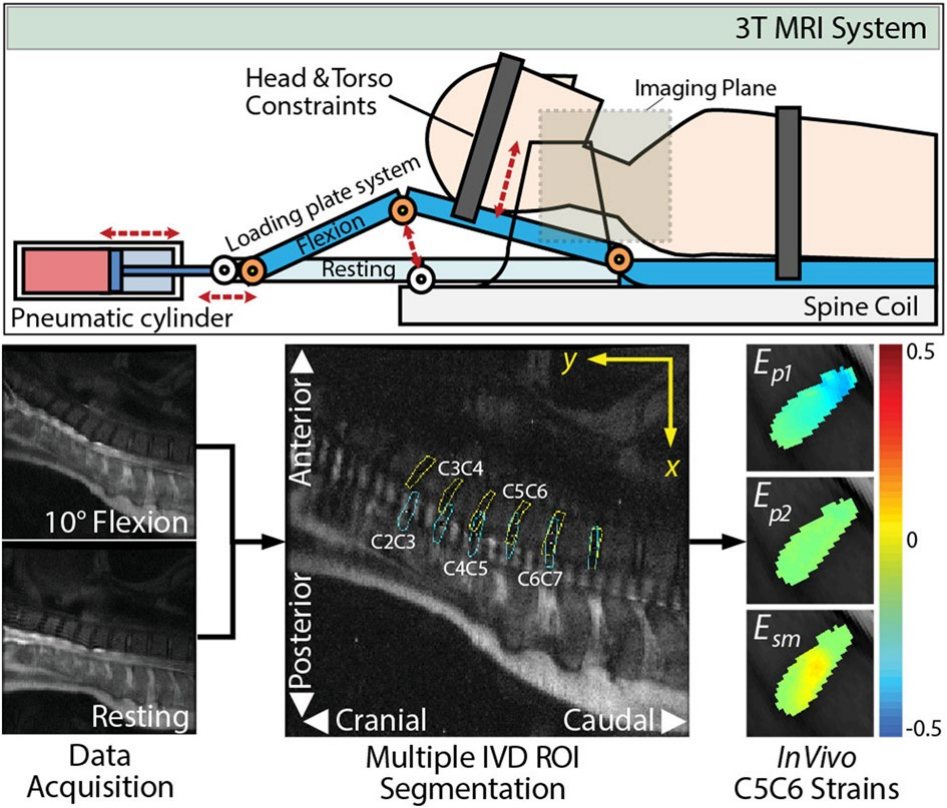
Synchronized bending of the neck with MRI acquisition enables measurement of intratissue motion and strain of adjacent intervertebral discs (IVDs) in vivo by dualMRI. An MRI compatible loading device consisting of a pneumatic cylinder and a two-bar linkage synchronized with DENSE acquisition leads to flexion–extension of the cervical and thoracic spine (C2C3–T2T3) in the sagittal plane. Cyclic IVD motion was acquired over 160 cycles, with the head held in a flexed (reference) state for 5.5 s, extension occurring in a transition period of 0.5 s, and a 2.0 s extension (deformed) state during which image acquisition occurred. An eight-channel spine coil enables cervical-thoracic intervertebral disc (IVD) acquisition at a neutral position and at 10° flexion. Region of interest (ROI) masks were manually segmented from images of the cervical spine with + *y* in the cranial direction and + *x* in the posterior direction. Voxelwise in vivo IVD Principal Strains (*E*_*p1*_, *E*_*p2*_) and Maximum Shear Strain (*E*_*sm*_) were calculated per IVD segment from the resultant displacement fields.

Voxelwise in vivo cervical and thoracic (C2C3-T2T3) IVD displacements and strains fields were computed via a cyclic 10° flexion–extension dualMRI protocol (Fig. 1) for fifteen healthy subjects (Table 1; M/F: 5/10, average age: 24.7 years, range: 20–29 years) with Institutional Review Board approval. Strain field regions-of-interest (ROIs) were segmented and analyzed for inter- and intra-disc differences. Relaxometry ROIs and dualMRI extrema were assessed for inter-disc differences and correlated.

**Table 1:** Basic demographic information about the male (*n* = 5) and female (*n* = 10) volunteers.

	Age (years)	Height (cm)	Weight (kg)
Males (*n* = 5)	24.2 (1.83)	177.2 (2.89)	68.4 (1.86)
Females (*n* = 10)	24.5 (1.42)	165.0 (1.87)	57.6 (1.96)

In this study, volunteers were approximately the same age (average age: 24.7, range: 20–29 years) regardless of sex with the males having a higher height and weight. Data is presented as mean (standard error of the mean).

### Precision of the cervical flexion–extension device

The cervical flexion–extension loading device precision was evaluated through the repeated testing of an MRI phantom under a similar cyclic loading protocol as the human subject experimental settings with ssFSE imaging. We found the 95% confidence interval (CI) repeatability error of displacement measurements were ± 0.31 mm and ± 0.35 mm for *x* and *y* shift respectively, both of which were nearly an order of magnitude lower than the voxel width/height (2.84 mm) (Supplementary Information).

### Intervertebral disc displacement and strain fields

**Figure 2:**
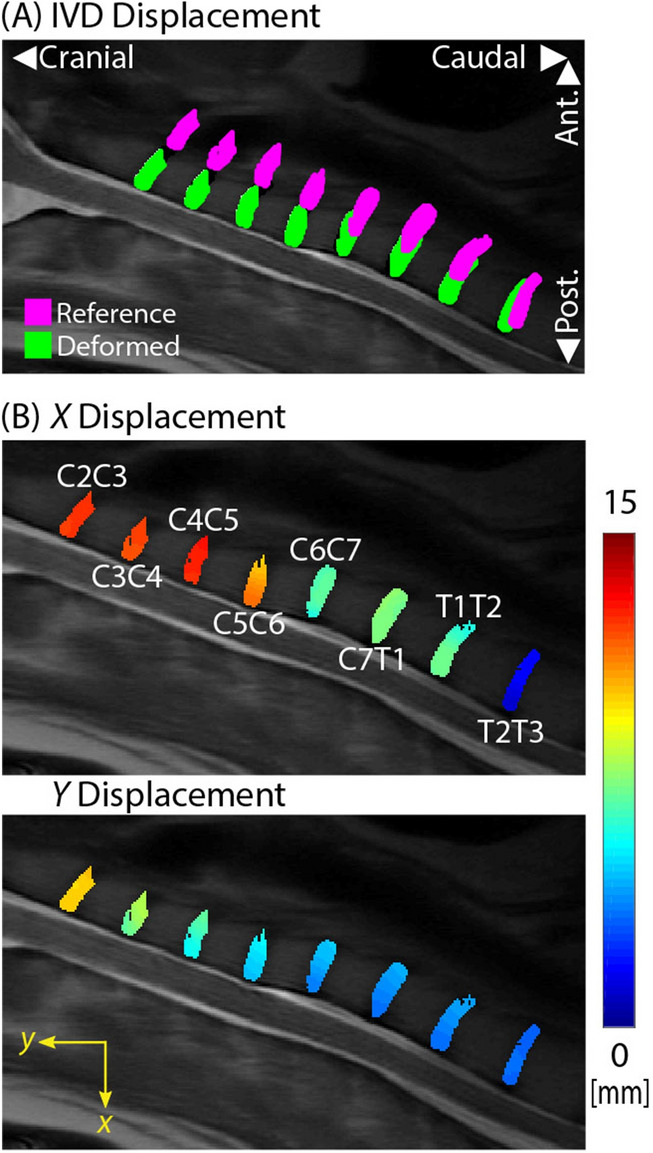
dualMRI enables in vivo imaging and calculation of intratissue displacements for multiple adjacent disc segments in a single subject. (**A**) IVD displacement is calculated from the flexed position (reference/magenta) to the neutral (deformed/green) IVD position with dualMRI. Positive *x* and *y* displacements are in the posterior and cranial directions respectively. (**B**) Smoothed *x* and *y* displacements of the cervical and thoracic IVDs (C2C3–T2T3) shows intratissue deformations not available with traditional methods, which commonly assess only bulk (e.g. rigid body) motion of the tissue. Both *x* and *y* displacements caudally decrease with higher displacements in the *x* direction, indicating bending motion in the sagittal plane.

Adjacent IVD displacements were calculated from the flexed position (reference/magenta) to the neutral position (deformed/green) utilizing dualMRI phase data (Fig. 2A). Smoothed *x* (range: − 2.22 mm to 15.15 mm) and *y* (range: − 2.36 mm to 13.43 mm) cervical and thoracic displacements were computed relative to the posterior and caudal directions respectively (Fig. 2B).

**Figure 3:**
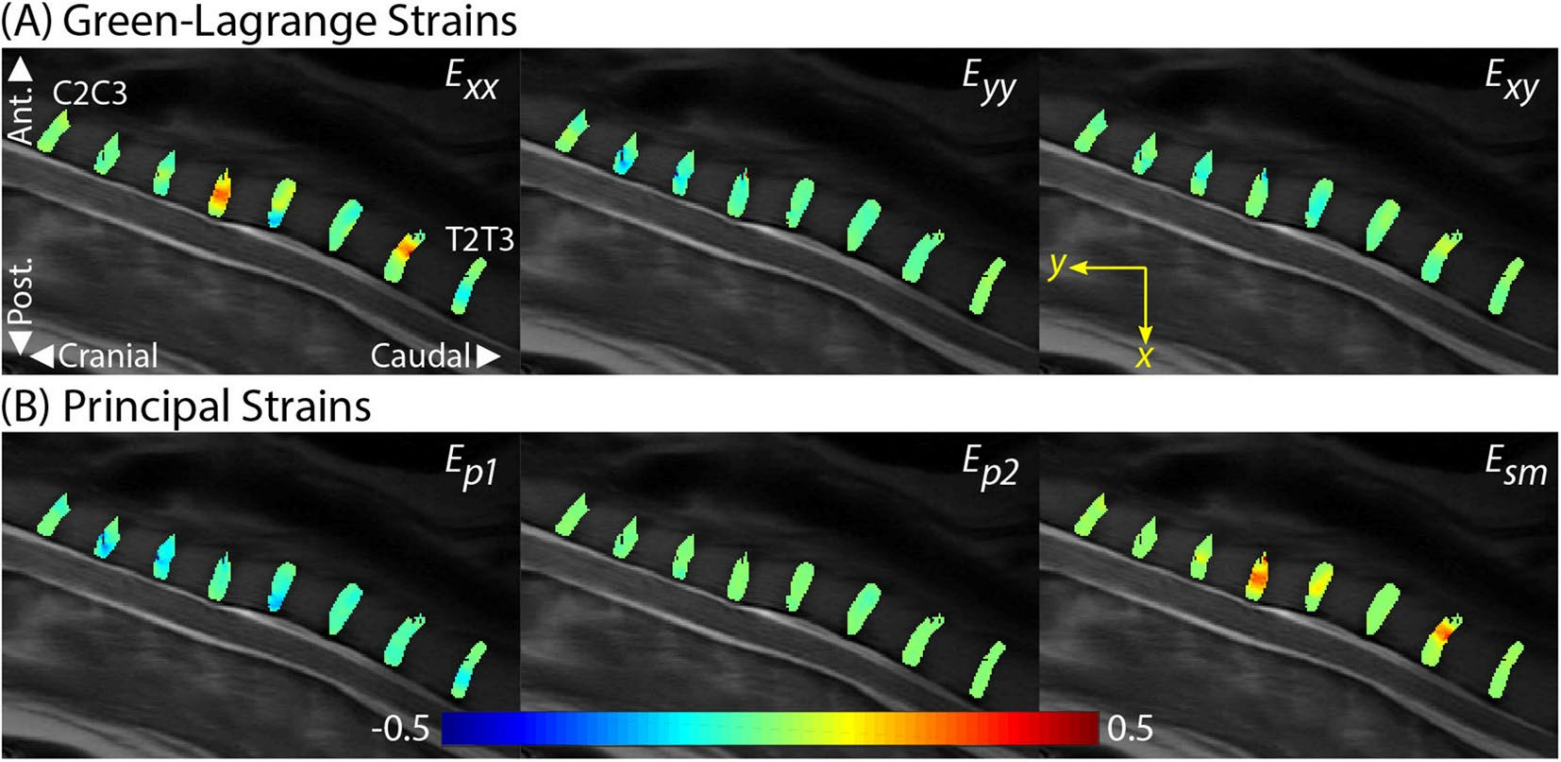
dualMRI facilitates the calculation of spatially complex Cartesian-based and principal direction strains on adjacent IVDs (C2C3–T2T3). (**A**) Green–Lagrange strains (*E*_*xx*_, *E*_*yy*_, *E*_*xy*_) were calculated at each voxel location from the smoothed displacement fields. Strains of increased magnitude were observed in the *E*_*xx*_ data. (**B**) Principal strains (*E*_*p1*_, *E*_*p2*_) and maximum shear strain (*E*_*sm*_) indicated spatial heterogeneity of magnitudes and demonstrated prominent patterns of elevated *E*_*p1*_ and *E*_*sm*_ strains.

Spatially complex Green–Lagrange (*E*_*xx*_, *E*_*yy*_, *E*_*xy*_), principal (*E*_*p1*_ and *E*_*p2*_*)*, and maximum shear (*E*_*sm*_) strains were calculated from the displacement fields (Fig. 3). Increased magnitudes (± 0.5) were observed in the *E*_*xx*_ strain fields (Fig. 3A). Heterogeneous IVD responses were observed in the first (maximum) principal strain (*E*_*p1*_ | range: − 20% to 0%) and *E*_*sm*_ (range: 0% to 37%) (Fig. 3B). The second (minimum) principal strain (*E*_*p2*_) exhibited more uniform spatial patterns with lower magnitudes (range: − 6% to 2%).

### Inter-disc spatial analysis

**Figure 4:**
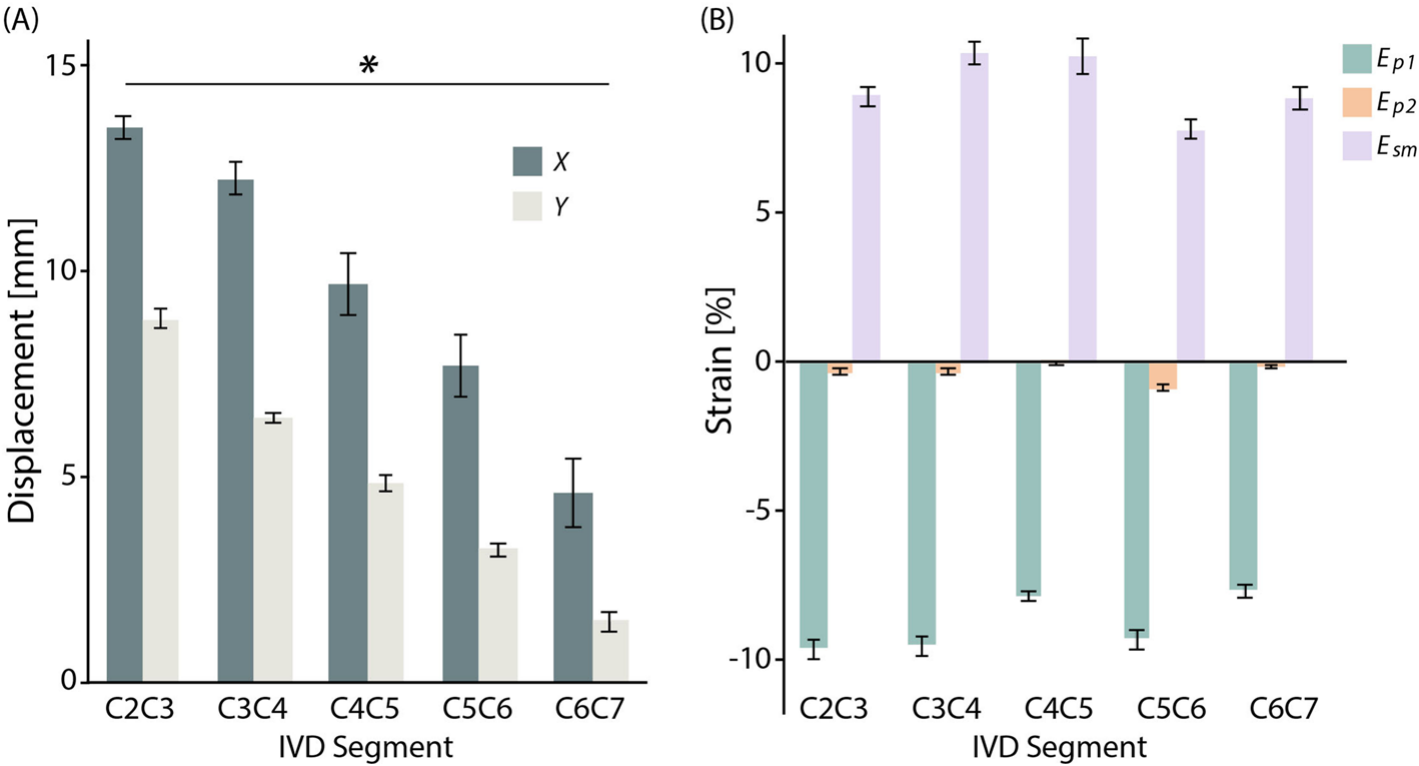
In our study cohort, inter-tissue displacements decreased in a cranio-caudal direction, while maximum principal strain (*E*_*p1*_) and maximum shear strain (*E*_*sm*_) maintained elevated intratissue strain magnitudes compared to minimum principal strain (*E*_*p2*_). (**A**) Population-level cervical IVD displacements by dualMRI significantly depended on position in respective *x* and *y* directions (*p < 0.01). (**B**) *E*_*p1*_ and *E*_*sm*_ approached or exceeded 10% in adjacent IVD segments, although adjacent-level strains were not significantly different (p > 0.01) in the cranio-caudal direction. Error bars represent standard error of the mean (SEM).

Inter-tissue displacements and strains (*E*_*p1*_, *E*_*p2*_, and *E*_*sm*_) produced via cyclic dualMRI depended on the IVD segment level (Fig. 4). IVD *x* and *y* displacements significantly correlated (p < 0.01) with IVD position (Fig. 4A) signifying sagittal plane movement. Elevated levels of *E*_*p1*_ and *E*_*sm*_, approaching 10% in adjacent IVD segments, were observed with minimal *E*_*p2*_ strains (Fig. 4B). However, significant level-dependent differences were not found (p > 0.01).

### Intra-disc spatial analysis

**Figure 5:**
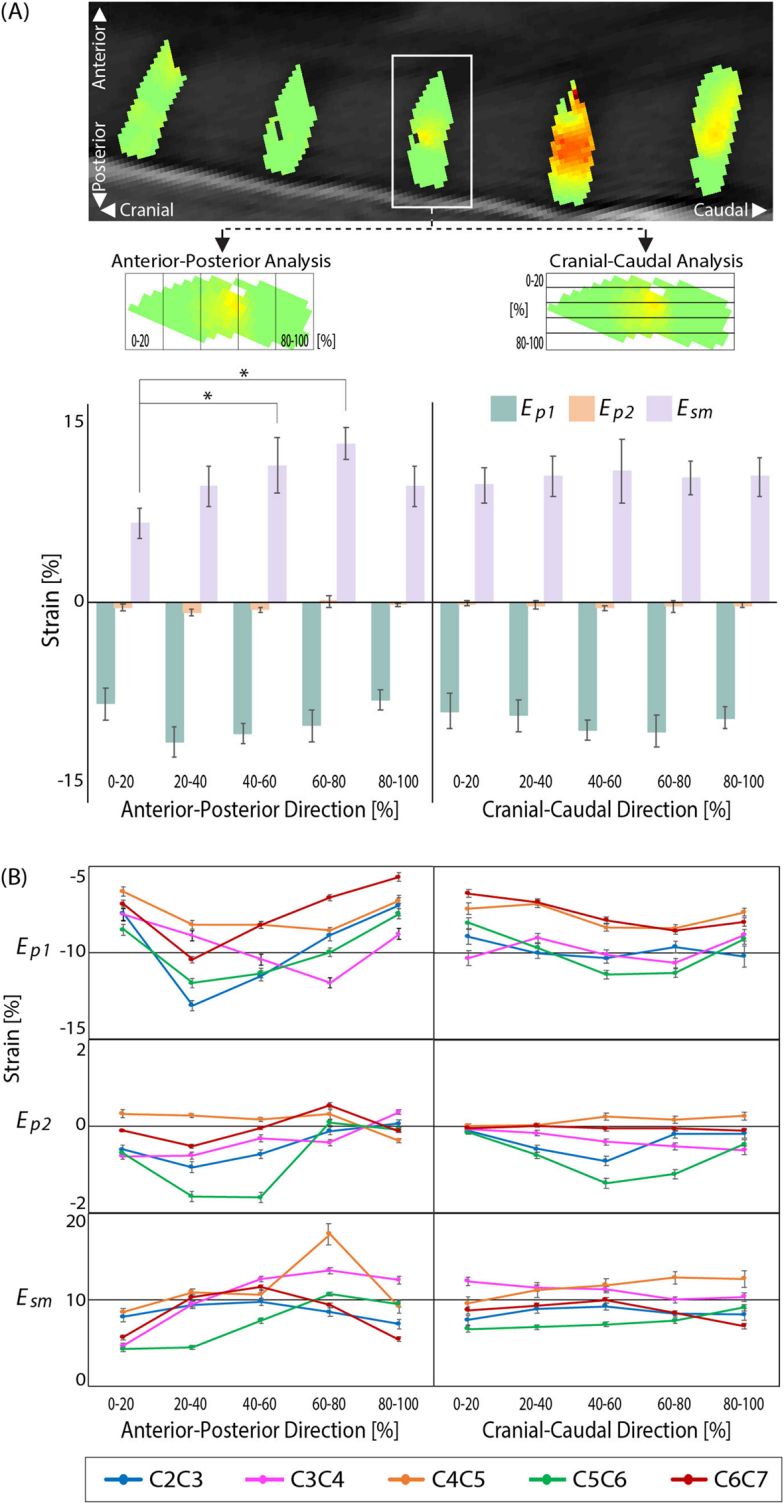
Within-disc analysis reveals increased *E*_*sm*_ in the posterior regions of the IVD. To perform intratissue regional analysis, each IVD strain field was spatially divided (binned) into five equal sections (~ 50 voxels each) in anterior–posterior and cranio-caudal directions and averaged per disc. (**A**) Cervical discs (C2C3–C6C7) *E*_*p1*_ strains spatially varied in the anterior–posterior direction while *E*_*p2*_ strain differences were minimal. *E*_*sm*_ strains were significantly increased (*p < 0.01) in the posterior (40–60% and 60–80%) locations compared to the anterior (0–20%) location. Analysis of the cranio-caudal strain regions revealed little variation (p > 0.01) for all strain measures (*E*_*p1*_, *E*_*p2*_, *E*_*sm*_). (**B**) The anterior–posterior and cranio-caudal segmentation of each disc separated by IVD indicates varying anterior–posterior responses in each IVD for all strains (*E*_*p1*_, *E*_*p2*_, *E*_*sm*_) while the cranio-caudal responses were more uniform. Error bars represent SEM.

Within-disc intratissue spatial analysis was performed by partitioning each cervical (C2C3–C6C7) IVD into five equally spaced sections (~ 50 voxels per section) in both anterior–posterior and cranial-caudal directions, and averaged per disc (Fig. 5). IVD section averages (Fig. 5A) revealed nonuniform strains for *E*_*p1*_ and *E*_*sm*_ in both directions while the *E*_*p2*_ responses were minimal. *E*_*p1*_ values were found to be section-dependent (p < 0.01) in both anterior–posterior and cranial-caudal directions, but with no pair-wise differences. *E*_*sm*_ values were significantly higher (p < 0.01) in posterior (40–60% and 60–80%) regions compared to anterior (0–20%) regions indicating an uneven anterior–posterior strain distribution on the IVD during extension. *E*_*p1*_ and *E*_*sm*_ strain trends were inverse of each other in the anterior–posterior direction. Significant differences were not found in directional *E*_*p2*_ spatial analyses. The separation of each strain by IVD revealed the respective strain contribution for each segment per direction (Fig. 5B). The anterior–posterior indicated varying responses per IVD while the cranio-caudal responses were observed to be more homogeneous.

### Relaxometry and dualMRI extrema

**Figure 6:**
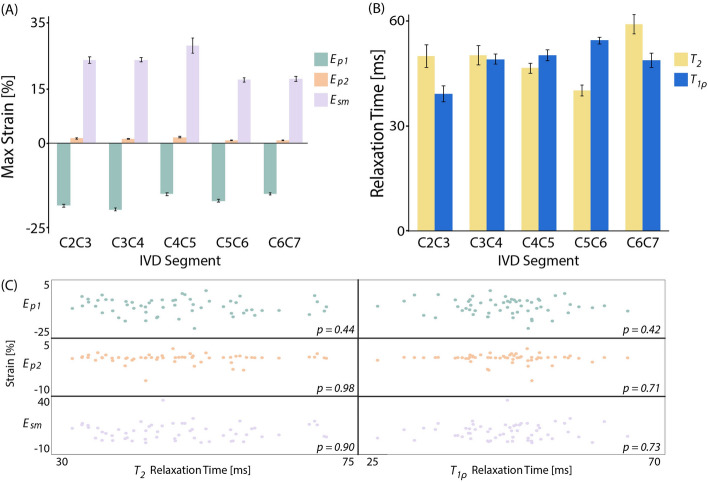
Relaxometry-based structure did not correlate with dualMRI-based mechanical function. (**A**) The absolute maximum 10% of each strain measure (*E*_*p1*_, *E*_*p2*_, *E*_*sm*_) represents the functional response extremes by each IVD to simple flexion–extension. The maximum strains revealed no significant differences across IVD segment (p > 0.01). (**B**) The average monoexponential qMRI (T_2_ and T_1ρ_) values were determined at each IVD segment as an indicator for spatial structural. Significant differences in relaxometry data were not found between adjacent discs (p > 0.01). (**C**) Correlations between whole disc strain and relaxation time were not significant (p > 0.01) for any combination (Strains: *E*_*p1*_, *E*_*p2*_, and *E*_*sm*_ | Relaxometry: T_2_ and T_1ρ_). The lack of correlation in a bulk disc analysis suggests both structural and functional data is needed to characterize the IVD in vivo. Error bars represent SEM.

Extrema in the functional IVD response were represented by maximum strain data, defined as the absolute maximum 10% of each metric (*E*_*p1*_, *E*_*p2*_, and *E*_*sm*_), and qualitatively corresponded with IVD level (Fig. 6A). However, level-wise significant differences were not found (p > 0.01). Notably, the maximum *E*_*sm*_ and minimum *E*_*p1*_ were at the same IVD location (C4C5).

Quantitative MRI (qMRI) relaxometry images were collected with the spine in the neutral (deformed) position prior to dualMRI acquisition as a measure of spatial structure^34–37^. Monoexponential relaxometry values averaged 48.8 ± 10 ms for T_2_ and 46.7 ± 7.1 ms for T_1*ρ*_. Significant differences between IVDs were not found for either relaxometry metric (*p* > 0.01) (Fig. 6B).

Correlations between the maximum strain values and qMRI data (Fig. 6C), a potential indicator of an MRI-based structural (qMRI) and functional (dualMRI) relationship, were not significant (p > 0.01) for any combination (strains: *E*_*p1*_, *E*_*p2*_, and *E*_*sm*_ | relaxometry: T_2_ and T_1ρ_). The lack of a bulk relationship between the whole disc structural and functional data highlights the inability of each to be used as a surrogate for the other.

## Discussion

This study utilized dualMRI for non-invasive in vivo measurement of IVD biomechanics (displacements and strains) in human volunteers. To our knowledge, this is the first study to analyze inter- and intra-IVD strain patterns in vivo at the voxel level. A custom 10° cyclic loading device for flexion–extension motion of the cervical spine was manufactured and coupled with optimized imaging parameters establishing the in vivo workflow. Prominent first (maximum) principal strain and maximum shear strain magnitudes found between discs with significant intratissue differences demonstrate the value added of this technique for functional data acquisition.

Intra-disc evaluation revealed heterogenous strain patterns providing insights into IVD load transfer and dissipation. The change in displacement magnitudes per IVD (Fig. 4A) indicated increased translation for each vertebra in the cranial direction leading to IVD strain response magnitudes in the anterior–posterior directions with more uniform cranio-caudal responses (Fig. 5B). The *E*_*sm*_ strain differences found between the anterior (0–20%) and posterior (40–60% and 60–80%) regions of the IVD (Fig. 5A) were likely due to the rigid constraints of the adjacent vertebrae. The homogeneity of all strains (*E*_*p1*_, *E*_*p2*_, *E*_*sm*_) in the cranio-caudal direction suggests the ability of the NP to evenly dissipate an applied load. The within-disc differences found in the spatial analysis highlight the intratissue analysis capability of dualMRI.

Computed average inter-tissue displacement and strain (*E*_*p1*_, *E*_*p2*_, *E*_*sm*_) patterns (Fig. 4) indicated level-dependent strain mitigation patterns in the cervical spine. The elevated *E*_*p1*_ strains can be related to the rigid body motion of the relatively stiff surrounding vertebrae. Cervical extension translates the vertebrae applying a load upon each cervical IVD resulting in IVD contraction along the sagittal plane (the imaging plane of this study), captured by *E*_*p1*_, and possible expansion along the out-of-plane (coronal) axis (not captured in the single-slice imaging plane of this study). Studying the inter-tissue variation of cervical IVD strain responses to different loading schemes (e.g. rotation, lateral flexion) would provide great insight into healthy and diseased disc biomechanical responses. Additionally, strain magnitudes presented here were greater than some prior ex vivo studies^32,33^, largely thought to be due to the change in loading mechanics (i.e. 445–450 N compression vs. 10° cervical flexion), but agree with mathematical models^38^. Further study of these strain magnitude discrepancies is an area of future research interest.

Acquisition of relaxometry (T_2_ and T_1ρ_) and dualMRI data in the same imaging session enables a direct comparison between relaxometry-based structural and dualMRI-based functional measures. Qualitative absolute maximum strain patterns (Fig. 6A) mimicked the average strain data (Fig. 4B) with undulating level-dependent magnitudes and a maximum *E*_*sm*_ and minimum *E*_*p1*_ at C4C5. qMRI metrics, T_1*ρ*_ in particular, have been shown to be sensitive to IVD integrity and altered biomechanics in studies focused on differences between healthy and diseased states^39,40^. The lack of quantitative and qualitative trends in the monoexponential relaxometry data (Fig. 6B) or in the correlations between whole disc dualMRI extrema and relaxometry (Fig. 6C) suggests the inability for each metric to serve as a surrogate for the other in healthy tissue, particularly at the level of interdisc comparisons. Capturing qMRI and dualMRI images in the same session enables the use of both metrics in tandem yielding further (structure–function) insights into the state of IVD health in vivo*.*

Intratissue spatial analysis of IVD biomechanical metrics, made possible by dualMRI, may elucidate mechanical alterations created by the spinal fusion of adjacent discs or total disc arthroplasty. Adjacent discs are likely to begin degenerating after spinal fusion surgery^41,42^ or total disc arthroscopy^43^ often requiring additional surgery. dualMRI enables a biomechanical evaluation of an entire spinal segment. Investigation into spinal fusion with dualMRI may further the understanding how a spinal fusion alters native tissue biomechanics in vivo and inform future surgical techniques and devices.

The C5C6 IVD has been shown to be particularly susceptible to damage from aging or traumatic event^44–46^. The C5C6 IVD average strain data demonstrated a local *E*_*p1*_ maxima and the absolute minimum *E*_*sm*_ (Fig. 4B). Furthermore, the adjacent C4C5 disc presented the 10% absolute maximum *E*_*sm*_ and minimum *E*_*p1*_ (Fig. 6A). The relative difference in healthy adjacent cervical disc strain patterns may be an indicator for the increased C5C6 IVD damage vulnerability. Notably, the observed strain pattern extrema do not agree with current mathematical models of acute trauma^38^. Further study of this disagreement, likely due to the differences in loading rates and magnitudes of the viscoelastic IVD, may improve future simulation accuracy.

**Figure 7:**
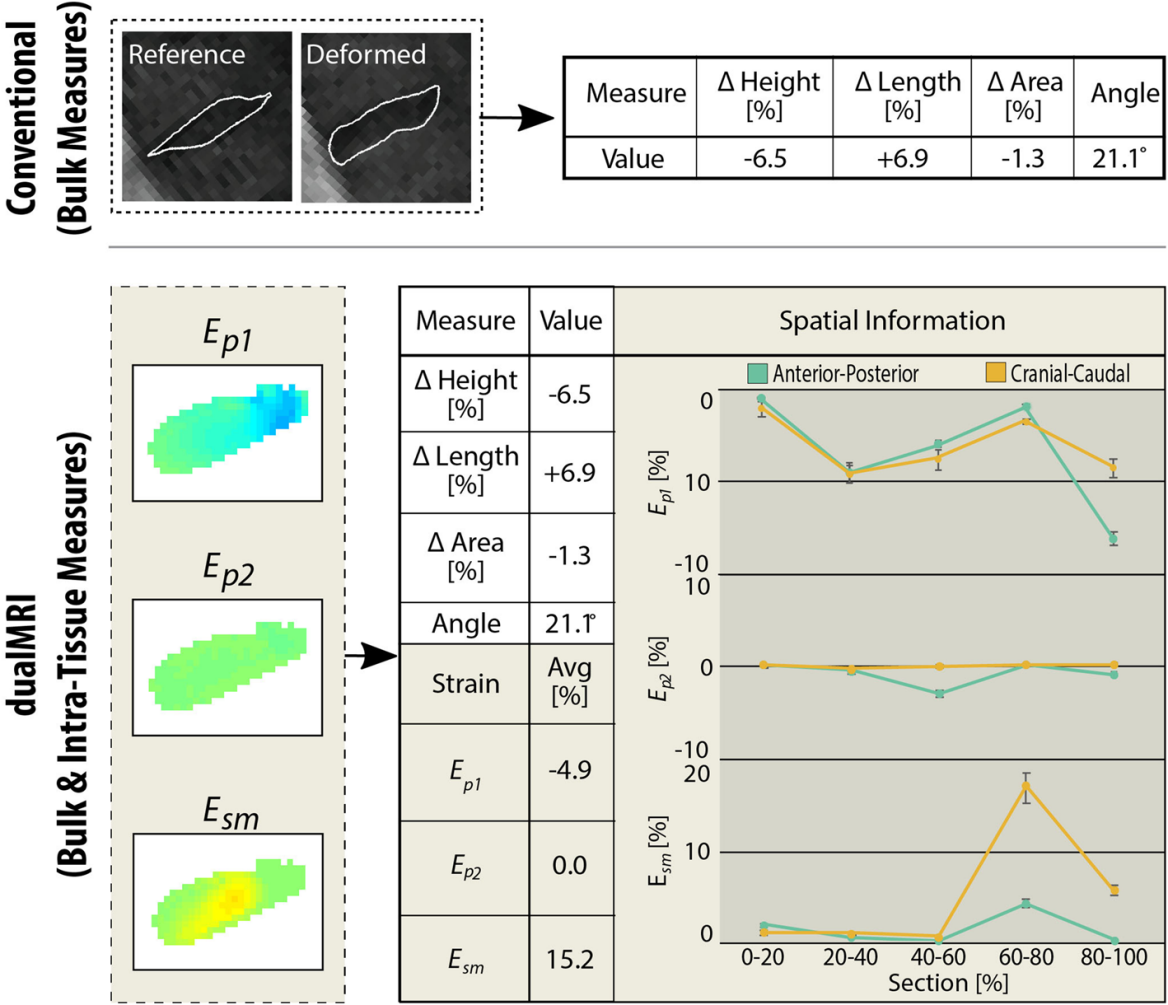
dualMRI allows for a more extensive and in-depth functional analysis compared to conventional morphology-based techniques. Conventional techniques calculate bulk differences (e.g. changes in height, length, area, and angle) to help indirectly inform or estimate functional measures. dualMRI allows for calculation of the same bulk measures while simultaneously providing intratissue biomechanical data. The calculation of principal strains (*E*_*p1*_ and *E*_*p2*_) and maximum shear strain (*E*_*sm*_) can be computed either as whole disc averages or by (spatially-dependent) section to allow for a more comprehensive biomechanical analysis.

dualMRI is advantageous over more conventional morphology-based techniques due to its voxel-level, in-plane measurement ability in vivo*.* Local isolated degeneration phenotypes^12,16^ and potential repair strategies^14,15^ necessitate direct intratissue biomechanical measurement. Intratissue strains of the IVD ex vivo have been reported via MRI^47,48^; however, the method used for these studies (i.e. direct compression of the spinal column) is not easily suitable for in vivo analysis. MRI has also been utilized in vivo to directly report bulk and regional mechanical changes^49,50^ (e.g. disc volume changes, disc height differences, etc.), as well as hydration variation^51^ (as a surrogate for mechanics), and to indirectly report IVD deformation in conjunction with 3D modeling^52^. Intratissue IVD strains have been reported indirectly with combinatorial methods (e.g. radiography and 3D modeling^53–55^ and fluoroscopy/MRI^56–58^) typically extrapolating displacement and strain fields from digital reconstruction of vertebral endplate movements. Nevertheless, to our knowledge, no in vivo studies report direct voxel-level intratissue strains from a single noninvasive modality, which demonstrates the improvement of dualMRI over more composite and conventional techniques (Fig. 7). Additionally, dualMRI allows for biomechanical calculations not previously possible (i.e. direct shear strain measurement)^59^ and is ideally situated for monitoring and treatment evaluation of IVDD (voxel-level region specific biomechanical alterations)^14,15^.

dualMRI may be more informative than other elastography techniques for IVD biomechanics. Optical Coherence Elastography (OCT), a common elastography technique, has excellent spatial resolution (5–15 µm)^60,61^. However, the low penetration depth of OCT^62^ and small FOV make it unsuitable for in vivo IVD assessment. MRE has been a developing field for assessing the biomechanics of the IVD in vivo^22,23,63^ as the shear modulus of the NP has been shown to increase by a factor of eight due to IVDD^64^. However, majority of these studies were only able to report parameters of the relatively pliable healthy NP, due to increased shear wave magnitudes and shear wave attenuation, leaving open the ability of MRE to easily resolve stiffness of the AF and diseased NP. Water et al. has circumvented the attenuation limitation for the larger lumbar discs reporting stiffnesses for the NP and AF and relating escalations in stiffness to increases in Pfirrmann score^23^. Unfortunately, the utilized principal frequency analysis only allows for a single measurement per ROI (e.g. NP or AF) which eliminates intradisc variation analysis, a potential disc integrity indicator. Ultrasound Elastography (UE) also relies on shear waves suffering from the same limitations as MRE. UE has been performed on IVDs in vivo^65^; yet, the calculation of material properties was not possible as many inverse methods for UE (and MRE) require assumptions of tissue homogeneity and isotropy which are violated by the IVD structure. dualMRI operates without shear wave propagation and consequently is not hindered by the same constraints as MRE, making it an ideal candidate to directly explore the anisotropic and heterogeneous biomechanical behavior of the IVD in vivo, and under physiologically-relevant loading and spatial resolution.

Solving limitations of this preliminary dualMRI protocol could further improve dualMRI acquisition. The use of dualMRI dictated the need for an MRI-compatible loading device (i.e. non-metal) which can precisely and repeatedly load the tissue in a controlled manner. The repeatability of the cervical loading device used in this study was quantified and found to have displacement errors less than one pixel (Supplementary Information). However, it was not customized to each patient. If larger spinal sections are considered in the future, alterations to the design (e.g. length, height, etc.) or custom-fabricating individual components could account for anthropomorphic variations thus minimizing displacement error. Further design alteration, such as lower placement of a loading plate system, could easily enable investigation of various (e.g. lumbar) spinal segments. Additionally, diurnal variation in IVD responses, which can have noticeable biophysical effects^66^, were not considered in this study but should be taken into account in future work. Loading of the tissue itself is constrained by the need to reach a quasi-steady load deformation for optimal image quality^67^. The time dependent response of the tissue to reach this quasi-steady state limits the loading frequency, restricting the types of loading studies that can be reasonably imaged with dualMRI.

Multiple possibilities to decrease scan time could be explored in order to utilize facility time effectively and maximize patient comfort. In this study, each scan session was 45 min and required ~ 200 cervical flexion–extension cycles to reach sufficient SNR (i.e. SNR > 3). While discomfort was not reported from the healthy volunteers, such a loading regime could be damaging to those with prior injury or surgery (e.g. spinal fusion). To further reduce scan time, faster acquisition sequences should be considered, in addition to alternative (e.g. spiral) k-space sampling.

The work presented here establishes dualMRI as an effective tool for noninvasive quantification of IVD displacements and strains in vivo. Significant differences were found for each displacement direction and in the anterior–posterior maximum shear strain spatial analysis; the latter of which is not possible to measure with conventional morphometric techniques. Resultant strains may help elucidate biophysical and biomechanical cellular level behavior yielding insight into in vivo loading and structural alterations (proteoglycan loss, regions of damage) potentially providing a valuable biomarker for early disease states. Additionally, the combination of dualMRI with conventional relaxometry measures, which alone are unable to identify the subtle morphological changes present in the earliest stages of IVDD, may be a more sensitive technique for investigating and monitoring IVD biomechanics than any single metric. We anticipate the use of dualMRI in cervical flexion–extension to be of interest to the musculoskeletal community as a means of improving existing IVD mathematical models, informing tissue engineering construct creation, evaluating adjacent IVD biomechanics and health in vivo, and assessing potential IVDD treatment efficacy.

## Methods

We utilized dualMRI (displacements under applied loading by MRI) to calculate spatial patterns of deformation (displacements, strain) in adjacent IVDs during simple neck flexion–extension. dualMRI combines a phase-contrast-based displacement encoding pulse sequence with exogenous loading, in this case cyclic flexion–extension of the cervical spine resulting from a custom pneumatic device. The dualMRI protocol spatially encodes phase shifts, which, upon imaging, allow for the computation of voxelwise displacements and strains. Here, relaxometry imaging was first collected followed by cyclic dualMRI in healthy subjects with Institutional Review Board approval from the Human Research Protection Program at Purdue University with informed consent. All methods were carried out in accordance with relevant guidelines and regulations. Regions of interest (ROIs) were segmented out for both relaxometry and dualMRI image sets, and spatially assessed for inter- and intra-IVD differences.

### Relaxometry acquisition

Prior to dualMRI acquisition, the loading plate system was set at the resting state where localizer, anatomical (multi-slice gradient echo), and single slice qMRI T_2_ (TE: 6.78, 13.97, 21.15, 42.72 ms) and T_1*ρ*_ (SLP: 500 Hz TSL: 1, 5, 20, 40, 60 ms) relaxometry (FOV: 270 × 270 mm^2^, Matrix: 256 × 128 pixels^2^, Slice Thickness: 4 mm, Views Per Segment: 64, TR 1.2 s, Number of Slices: 26, ARC Acceleration Factor: 2) images were acquired.

Monoexponential qMRI maps were created by voxelwise decay curve fitting utilizing MATLAB’s curve fitting toolbox. Pixel decays with an R^2^ value < 0.66 or with an outlier decay time (Grubb’s Test, α = 0.05) were excluded from the study in order to minimize contributions by voxels with low signal quality^68^. Upon completion, the qMRI maps were smoothed with a locally weighted scatterplot smoothing (LOWESS) filter (span: 10 voxels) for noise minimization^69^.

### Cyclic cervical flexion–extension device design

An MRI compatible 2-bar linkage loading plate system was constructed to achieve cyclic cervical spine flexion–extension of 10°, a limit of space constraints within the MRI scanner and well inside the cervical column range of motion^52^ (Fig. 1). The base of the neck was aligned at the pivot point of the first bar linkage with the base of the apparatus while the subject’s forehead and torso were constrained with soft straps to minimize extraneous movement. Motion was accomplished with a computerized double-acting pneumatic cylinder attached at the opposite end of the linkage allowing control of speed and degree of flexion-extension^28^. While both patient and cervical flexion–extension device were inside a clinical 3.0 T MRI (General Electric Signa HDx, Waukesha, WI) system, a radio frequency spinal coil (General Electric 8-channel phase array) was placed directly under the flexion–extension device covering the length of the cervical and thoracic spine to enable imaging.

### Precision of the cervical flexion–extension device

The precision of the cervical flexion–extension system was quantified by calculating the displacement of a silicone phantom (Sylgard 527, Dow Corning, Elizabethtown, KY; dimensions: *l* × *w* × *t* = 23 × 10 × 4 mm^3^) under a similar cyclic loading protocol as the human subject experimental settings with ssFSE imaging. The flexion–extension protocol was repeated five times in series over three separate sessions.

To simulate the change of subjects, a 1 h break was taken between sessions during which the MRI bed was returned to the home position where the phantom was removed from the loading device and subsequently returned to its previous position using registration markers. The resultant displacements were then averaged and evaluated for precision (Supplementary Information).

### dualMRI acquisition

Displacement-encoded MRI was accomplished via a DENSE (displacement encoding with stimulated echoes) pulse sequence combined with a ssFSE (single-shot fast spin echo) sequence^32,70^ synchronized with pneumatically-actuated cyclic flexion–extension of the neck. The DENSE signal was encoded into the tissue during flexion (reference state) while the ssFSE read-out was performed in the resting (deformed) state. The use of the flexed position as the reference state increased the SNR (over × 4 greater) by allowing for a reduced transition period (1.25–0.5 s) minimizing signal decay in the tissue. DENSE displacement encoding was completed with a 0.33 π/mm encoding gradient. A 0 π/mm encoding gradient was collected as a phase reference map to eliminate unanticipated phase artifacts. DENSE phase map acquisition was performed in both anterior-posterior (*x*-axis) and cranio-caudal (*y*-axis) directions. Each encoding was phase cycled (± co/sine) to reduce artifacts due to a ssFSE readout error^71^.

Displacements ($\Delta x$ or $\Delta y$) were computed from the phase component ($\Delta \varphi$) of the MRI data in their respective directions relative to the T1T2 disc (assumed to be 0 displacement) by the following equation (shown here in the *x*-direction):

1 $$\Delta \varphi = \gamma_{H} t_{enc} (G_{de} - G_{de}^{^{\prime}} )\Delta x$$

where $\gamma_{H}$ is the gyromagnetic ratio of the ^1^H proton, $t_{enc}$ the encoding duration, $G_{de}$ the displacement encoded gradient magnitude, and $G_{de}^{^{\prime}}$ the reference map. The resultant displacement maps were smoothed with 100 rounds of 5 pixel × 5 pixel Gaussian kernel filtering with a bisquare function to improve robustness^24,72^. Green–Lagrangian (*E*_*xx*_, *E*_*yy*_, and *E*_*xy*_) and principle strains (*E*_*p1*_ and *E*_*p2*_) as well as maximum shear strain (*E*_*sm*_) were calculated from the smoothed displacement images using custom code^24,25,28,32,33^ (MATLAB, Mathworks, Natick, MA).

### In vivo dualMRI of human intervertebral discs

Potential subjects were pre-screened for prior neck or back injuries through interview and those with signs of asymptomatic morphological abnormalities in the IVD during the preliminary MRI examinations were excluded. Twenty healthy subjects (M/F: 10/10) were originally enrolled; However, five subjects were excluded from the study due to excessive noise in the MRI scans. The remaining fifteen healthy subjects (Table 1—M/F: 5/10, average age: 24.7, range: 20–29 years) completed successful imaging of cervical and thoracic IVDs (C2C3–T2T3).

To minimize inter-subject variability and establish a consistent focal point of bending about the C7T1 region, fluid capsules served as fiducial markers—one placed onto the hinge of the loading platen and the other marker placed on the C7 spinous processes. The subject was adjusted as necessary until the two markers were in a maximum proximity and within the localizer sequence FOV. To minimize body movement, subjects breathed and swallowed in sync with the non-acquisition period (4 s of the 8 s loading cycle).

A single loading sequence consisted of a flexed state time of 2 s, a transition period of 0.5 s, and a resting state of 5.5 s. 20–30 preconditioning load cycles were performed to minimize viscoelastic creep artifacts and allow the subject to adjust to the loading cycle regime.

After the preconditioning cycles, the dualMRI sequence was performed. For a single two-dimensional (2D) strain analysis, a total of 16 acquisitions were made: 2 DENSE phase maps × 4 phase cycling × 2 read-out directions. To achieve a sufficient signal to noise ratio (SNR) (i.e. ≥ 5)^28^, nine additional repeating acquisitions were obtained (i.e., NA = 10) resulting in 160 total loading cycles per subject. ssFSE parameters were: TE/TR = 72/5000 ms, mixing time = 500 ms, phase matrix size = 512 pixels × 512 pixels^2^, spatial resolution = 0.53 × 0.53 mm^2^, slice thickness = 7 mm. Total scanning time was approximately 45 min per subject.

### Relaxometry and dualMRI region of interest analysis

Whole disc ROIs were manually segmented from ssFSE images for all cervical discs (C2C3 to approximately T2T3, depending on individual anatomy). Relaxometry ROIs were segmented separately due to slight subject movement between relaxometry sequences and dualMRI. Discs with low SNR (< 1) were removed.

While dualMRI can be utilized on any spinal segment, the cervical spine is a distinct section that when flexed, as in this experimental setup or during whiplash, is likely to experience elevated magnitudes of strain and/or injury^38,44^. Therefore, analysis was focused on the cervical spine to specifically evaluate strains during simple flexion–extension (i.e. neck bending).

For bulk analysis, the displacement, strain, and relaxometry values for the ROIs were averaged for each IVD and then averaged across all participants (Figs. 4, 6). The absolute maximum strains were defined as the top 10% of strain magnitudes for each IVD and then averaged in the same manner (Fig. 6A).

Spatial analysis (Fig. 5A) was performed similarly. Each disc was rotated until its major axis angle was 0° with respect to the longitudinal (*x*) axis. Subsequently, each disc was spatially separated into five equal sections in both the anterior–posterior and cranio-caudal directions (~ 50 voxels per section). Voxel values were combined for all sections and then averaged.

### Statistics

Data sets were evaluated for normality by a Shapiro–Wilks test. Sets which failed a Shapiro–Wilks test were transformed to meet the normality assumption by using either a square root transformation, in the case of non-negative data sets, or the following equation for data sets with negative numbers:

2 $$Val_{New} = \sin \left( {Val} \right) \times \log \left( {\left| {Val} \right|} \right)$$

Displacement and strain were analyzed via a random mixed effects linear model with a type III sum of squares ANOVA treating patients as a random variable to determine inter- and intra-IVD differences. The relationship between strain and relaxometry data (Fig. 6C) was evaluated by linear regression. Significance for all tests was set at p < 0.01.

## Supplementary information

Supplementary Information.
