# Supplementary material for: In vivo intervertebral disc deformation: intratissue strain patterns within adjacent discs during flexion–extension
Source: Sci Rep. 2021 Jan 12;11:729. doi: 10.1038/s41598-020-77577-y (PMC7804136; doi:10.1038/s41598-020-77577-y)
Supplement: Supplementary file 1 — Supplementary Information. [file 41598_2020_77577_MOESM1_ESM.pdf]

SUPPLEMENTARY INFORMATION

For

In Vivo Intervertebral Disc Deformation: Intratissue Strain Patterns within Adjacent Discs During Flexion-Extension

Robert L. Wilson, M.S.[1], Leah Bowen, M.S.[1], Woong Kim, Ph.D.[2], Luyao Cai, Ph.D.[2], Eric A. Nauman, Ph.D.[3] Corey P. Neu, Ph.D.[1,2]

[1]Department of Mechanical Engineering
University of Colorado Boulder
1111 Engineering Drive, 427 UCB
Boulder, CO 80309

[2]Weldon School of Biomedical Engineering
Purdue University
206 S Martin Jischke Drive
West Lafayette, IN 47907

[3]School of Mechanical Engineering
Purdue University
585 Purdue Mall
West Lafayette, IN 47907

Table of Contents:

1. Precision of the Cervical Flexion-Extension Device
    1.1. ssFSE Image Collection
    1.2. Image Processing and Analysis
    1.3. Cervical Flexion-Extension Loading Precision Results

1. Precision of the Cervical Flexion-Extension Device

1.1 ssFSE Image Collection

Quantification of the cervical flexion-extension loading device precision was completed using a fabricated MRI phantom imaged in series over multiple sessions. A transparent MRI phantom (Sylgard 527, Dow Corning, Elizabethtown, KY; LWT dim: 23×10×4mm) embedded with Delrin beads of varying diameters (1/4", 1/8", 3/32") was constructed (Supplemental Figure 1 (A-B)) and secured into the custom cyclic cervical flexion-extension device (Supplemental Figure 1 (C-D)).

The flexion-extension device precision was measured by cyclically bending the phantom (Supplemental Figure 1 (E-F)) and obtaining ssFSE scans in the deformed position five times (Time between scans: 5s, Flexed position time: 1.25s, FOV: 180×180mm^2 [512pixels×512pixels], Resolution: 2.844mm/pixel) in series over three separate sessions with eight ssFSE images per protocol resulting in 120 scans (5 imaging protocols × 3 imaging sessions × 8 scans per protocol).

To simulate the change of subjects, a 1hr break was taken between sessions with the MRI bed returned to the home position and the phantom removed from the flexion-extension device. After the break, the phantom was returned to its previous position using a gel capsule marker.

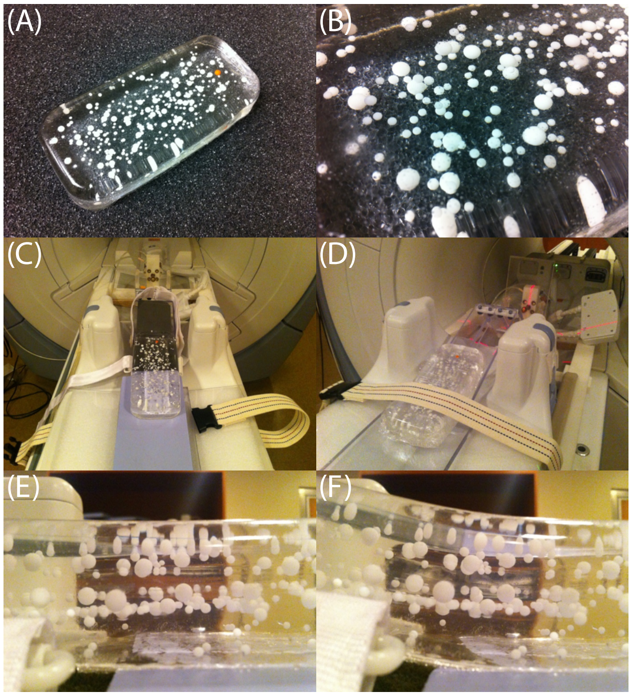

Supplemental Figure 1: An MRI phantom was constructed and utilized for the evaluation of the flexion-extension device precision by cyclically deforming the sample multiple times over a series of ssFSE imaging sessions. (A) The transparent MRI phantom enabled the tracking of multiple Delrin beads to accurately assess the loading precision of the flexion-extension device. (B) An enhanced view of the MRI phantom highlights the different sized embedded Delrin beads used for displacement registration. (C) The MRI phantom placed into the MRI scanner with the loading device underneath. (D) A closer view of the MRI phantom strapped into the flexion-extension device. (E) An undeformed view of the phantom in the loading apparatus. (F) A deformed view of the phantom being deformed by the applied load of the pneumatic cylinder.

1.2 Image Processing and Analysis

ssFSE images were analyzed to quantify the cervical flexion-extension device displacement precision. Of the 120 scans preformed, 113 imaged were successfully acquired (Supplemental Figure 2A) and seven mis-fired as characterized by significant deviations from the normal trigger pulse, thought to be due to device overcharging or overheating (Supplemental Figure 2 (B-C)).

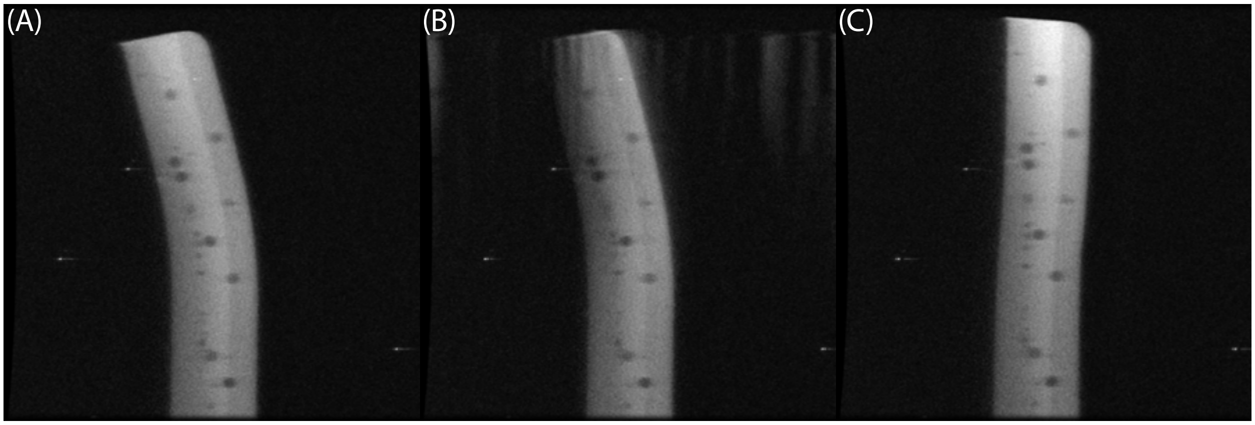

Supplemental Figure 2: The scanned image variations during image acquisition demonstrates a repeatable deformation process with minimal ssFSE acquisition misfires. (A) A representative successful image (n = 113) of the MRI phantom bending in the sagittal plane due to the flexion-extension device with high visibility of the embedded Delrin beads in various sizes. (B) Motion artifacts in ssFSE scans were a result of a misfire in acquisition with the ssFSE image being taken prematurely (n = 2). (C) A misfire in image acquisition caused scans to be captured prior to deformation by the flexion-extension device (n = 5).

Utilizing ImageJ (NIH, Bethesda, Maryland) in conjunction with an image stabilizer plug-in[1], ssFSE images were ridge-transformed via the Lucas-Kanade algorithm to a reference image (i.e. the first scanned image for each imaging session | n = 3) and subsequently evaluated for their x and y shift from their respective reference image (n = 110) (Supplemental Figure 3).

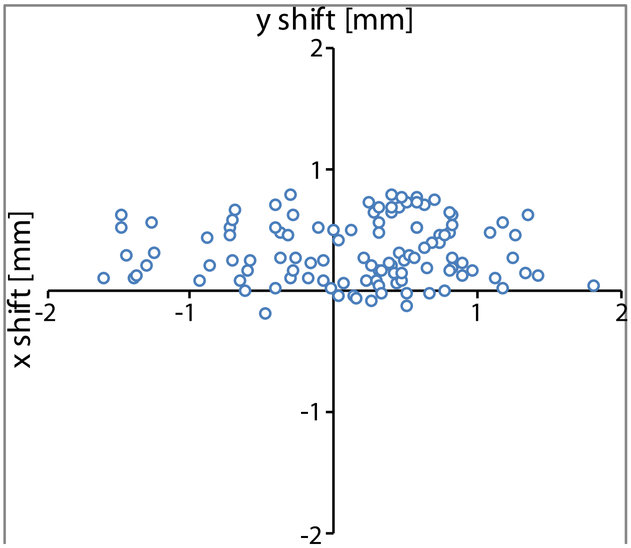

Supplemental Figure 3: The analysis of the x and y displacement shifts demonstrated the high precision of the cyclic flexion-extension device. Position shifts for all image samples in both directions were less than a single pixel (2.844mm) compared to the reference (n = 110).

1.3 Cervical Flexion-Extension Loading Precision Results

The resultant image variation analysis demonstrated minimal displacement repeatability error in the cervical flexion-extension loading apparatus. The *x* and *y* shift mean errors (n = 110) were 0.17mm and 0.30mm respectively with upper 95% confidence interval (CI) limits of ±0.31mm and ±0.35mm. (Supplemental Table 1). Even at the 95% CI, the sample variability was considerably less than the resolution of a single pixel (2.844mm) thus yielding an acceptable level of precision.

Supplemental Table 1: The summary of displacement variability in the dualMRI process. As evidenced by displacement error means and 95% confidence intervals (bolded) in both directions (*x* and *y*) lower than a single pixel (2.844mm), the cyclic cervical flexion-extension device was sufficiently precise.

	***x* shift [mm]**	***y* shift [mm]**
**Mean**	**0.1732655**	**0.3015519**
Std Err of the Mean	0.0682164	0.0237707
**Upper 95% Mean**	**0.3084275**	**0.3486504**
Lower 95% Mean	0.0381035	0.2544534
n = 110		

References:

1. K. Li, "The image stabilizer plugin for ImageJ, "http://www.cs.cmu.edu/~kangli/code/Image_Stabilizer.html, February 2008.
